# Identification of the gut microbiota affecting *Salmonella pullorum* and their relationship with reproductive performance in hens

**DOI:** 10.3389/fmicb.2023.1216542

**Published:** 2023-07-27

**Authors:** Qing Niu, Xiaoxu Wang, Xinyong Qi, Changjian Cao, Kaixuan Yang, Caiju Gu, Zhenxiang Zhou, Qizhong Huang

**Affiliations:** ^1^Animal Husbandry and Veterinary Research Institute, Shanghai Academy of Agricultural Science, Shanghai, China; ^2^Shanghai Animal Disease Control Center, Shanghai, China; ^3^Shanghai Runzhuang Agricultural Technology Limited Liability Company, Shanghai, China

**Keywords:** gut microbiota, poultry, pullorum, microbial structure, prediction functions

## Abstract

**Introduction:**

Pullorum disease is one of the common bacterial infectious diseases caused by *Salmonella pullorum* (*S. pullorum*), which can result in a decrease in the reproductive performance of laying hens, thus causing considerable economic losses. However, studies about the characteristics of intestinal microbiota with pullorum and their potential association with reproductive performance in hens are still limited. This study was to identify the gut microbiota associated with *S. pullorum* in poultry.

**Methods:**

A total of 30 hens with *S. pullorum*-negative (PN) and 30 hens with *S. pullorum*-positive (PP) were analyzed for hatching eggs laid in 2 weeks (HEL), fertilization eggs (FE), chick number (CN), and microbial structure.

**Results:**

There were significant differences in HEL (*p* < 0.01), FE (*p* < 0.01), and CN (*p* < 0.01) between PP and PN. Histomorphological observations showed abnormal morphology of the ovaries and fallopian tubes and low integrity of epithelial tissue in the ileum and cecum in PP. 16S rRNA gene sequencing revealed that beneficial cecal microbes, such as *Bacteroides, Desulfovibrio*, and *Megamonas*, were positively correlated with reproductive performance and had lower abundance in PP (*p* = 0.001). Furthermore, diminished phosphotransferase system (PTS) and pentose phosphate pathway, butanoate metabolism and oxidative phosphorylation were also found in PP.

**Discussion:**

Taken together, this study clarified the morphological characteristics of the reproductive tract and intestines of chickens infected with *S. pullorum* and preliminarily explored the potential association between cecal microbiota and reproductive performance in hens. Our data may provide a reference for revealing the intestinal microbial characteristics of hens in resisting pullorum and exploring novel approaches to infection control in future studies.

## Introduction

Pullorum disease, caused by *Salmonella pullorum* (*S. pullorum*), is transmitted both vertically and horizontally in chickens (Berchieri et al., 2001; Li et al., 2018; Zhou et al., 2022). It is an acute systemic disease and is more common in young birds (Soria et al., 2012; Wang et al., 2020), and some of the infected adult birds are asymptomatic carriers that transmit the bacteria to the offspring and other chickens in the flock, some of which show the symptoms of diarrhea, decreased fertility and laying, reproductive tract abnormalities, inappetence, and weight loss (Shivaprasad, 2000; Wigley et al., 2001; Setta et al., 2012; Ding et al., 2021; Shen et al., 2022). Every year, *Salmonella* infection leads to serious economic losses in the poultry industry, especially in developing countries (Wigley et al., 2002; Barrow and Freitas Neto, 2011; Li et al., 2018, 2019). Previous studies suggested that in mainland China, PD was highly prevalent in the autumn, followed by the winter. Their findings also demonstrated that PD still posed a major threat to the poultry industry and that comprehensive and stringent strategies should be used to prevent and control this disease (Lv et al., 2022; Jiang et al., 2023). There is conclusive evidence that increased stocking density and larger farms result in an increased occurrence, persistence, and spread of *Salmonella* in laying hen flocks (Hazards et al., 2019). It causes decreased production performance and even the death of poultry, as well as being a threat to public health. Although eradication programs have been carried out as a prevention and control measure, the agglutination test results are erratic, including false-negative reactions and a lack of sensitivity, so *Salmonella* infection is still one of the most important problems worldwide (Barrow et al., 2012; Wang et al., 2020).

The use of antibiotics to prevent and treat bacterial diseases, such as *Salmonella*, leads to an increase in multiple drug-resistant bacteria worldwide (Zhou et al., 2020). Sulfonamides have been used in the treatment of pullorum disease, including sulfadiazine, sulfamerazine, sulfathiaole, sulfamethazine, and sulfaquinoxaline. However, most studies have indicated that no drug or combination of drugs has been found to be capable of eliminating infection from treated flocks (Shivaprasad, 2000). Globally, the animal industries are moving toward restricting and eventually a total ban on the usage of antibiotic growth promoters (Liao and Nyachoti, 2017). Meanwhile, the Chinese government is promoting a reduction in antibiotic use currently (Zhang et al., 2015). This trend prompts people to actively seek an ideal alternative to antibiotics.

The inclusion of alternative feed additives in lieu of antibiotics in animal diets is definitely required to support a profitable and sustainable poultry industry (Liao and Nyachoti, 2017). As is known, the gut microbiota can be manipulated by feed additives such as exclusion products, probiotics, prebiotics, organic acids, plant extracts, essential oils, and feed enzymes (Shin et al., 2008; De Lange et al., 2010; Le Bon et al., 2010; Heo et al., 2013). However, there is no report on preventing and controlling pullorum disease in the Chinese local chicken breeds. An animal's character can be affected by changes in the microbiota. As it is an opportunistic pathogen, the occurrence of pullorum disease is mainly caused by an imbalance of the intestinal microbiota (Shivaprasad, 2000). Probiotics are defined as a microbial feed supplement that beneficially affects the host animal by improving its intestinal balance (Fuller, 1989). They are a category of feed additives that can be used to replenish the gut microbial population while recuperating the host immune system (Liao and Nyachoti, 2017). Many recent studies have shown that humans and animals fed probiotics have altered intestinal microbiota, increased intestinal immunity, improved resistance to disease, reduced shedding of pathogens and disease symptoms, and improved health status (Zhao et al., 2013; Chiu et al., 2014; Upadhaya et al., 2015; Gao et al., 2017; Liao and Nyachoti, 2017; Li et al., 2017; Chen et al., 2020; Wang et al., 2021). The gut microbiota affecting the pullorum and its functions in chickens need further study.

Due to the large diversity of bacterial species, the gut microbiome contains circa 9 million unique protein-coding genes, but they have not been studied extensively (Rosenberg and Zilber-Rosenberg, 2018). The availability of high-throughput sequencing will shortly enable the sequencing of whole bacterial populations, enabling a more comprehensive view of bacterial evolution among related bacterial species (Barrow and Freitas Neto, 2011). This study aimed to analyze the intestinal microbial characteristics of hens resisting pullorum and their relationships with hatching eggs laid in 2 weeks (HEL), fertilization eggs (FE), and chick number (CN) in hens.

## Materials and methods

### Ethics statement

All procedures and the use of animals were carried out in accordance with the Guidelines for the Ethics and Animal Welfare Committee of the Shanghai Academy of Agricultural Sciences (No. SAASPZ0522051).

### Animal experiment and sample collection

*S. pullorum* infections in chickens were diagnosed by *S. pullorum* and *S. gallinarum* polyvalent antigen rapid slide agglutination test reagents (Beijing Zhonghai Biotech Co., Ltd., China). On a clean glass slide, 50 μl of polyvalent antigen and 50 μl of venous blood were placed. The samples were deemed positive if 50% or more agglutination occurred in the mixture within 2 min, and samples without agglutination were considered negative. In this study, two chicken groups were obtained after three times of the slide agglutination tests, and the results of the *S. pullorum* infection texts were positive or negative all three times. A total of 60 New Pudong chickens [samples from 30 hens with *S. pullorum*-negative (PN) and 30 hens with *S. pullorum*-positive (PP)] were selected from the experimental farm of the Shanghai Academy of Agricultural Sciences, Shanghai, China. These 60 hens were artificially inseminated at the age of 47 weeks. Recording the number of eggs laid, fertilized, and hatched chicks per hen and the performance, including HEL, FE, and CN, were analyzed during chick hatching processes. All eggs received disinfection before hatching. All hens were in the same poultry house (PN hens were distributed in the east of the poultry house and PP hens were distributed in the west of the poultry house), selected according to a unified breed standard, and fed antibiotic-free corn-soybean diets (Supplementary Table S1). Antibiotics in the feed or for any therapeutic purposes were not provided for hens after the age of 1 week. These 60 hens were slaughtered at the age of 49 weeks. After slaughter, tissue samples of the ovaries, fallopian tubes, ileum, and cecum with the size of about 2 × 3 cm were cut with a sterile scalpel and quickly stored in 4% paraformaldehyde for separate morphological observation. Cecal content was individually collected in 2 ml centrifuge tubes for 16S rRNA gene sequencing. All samples were kept in an ice box for preservation and transportation and then stored at −80°C in the laboratory (Janssen and Kersten, 2015).

### Morphological observation of the ovaries, fallopian tubes, ileum, and cecum

Tissues were routinely embedded in paraffin wax blocks, sectioned at 5 μm thickness, mounted on glass slides, and stained with hematoxylin & eosin (H&E). Morphological observations were conducted by a Nikon ECLIPSE 80i light microscope with a computer-assisted morphometric system (Nikon Corporation, Tokyo, Japan).

### 16S rRNA sequencing and bioinformatics analysis

The gut microbiota population in the hens with *S. pullorum*-negative (*n* = 30) and *S. pullorum*-positive (*n* = 30) was analyzed by 16S rRNA gene sequencing, respectively. Microbial community genomic DNA was extracted from cecum samples using the E.Z.N.A.^®^ soil DNA Kit (Omega Bio-tek, Norcross, GA, United States) according to the manufacturer's instructions. The DNA extract was checked on 1% agarose gel, and the DNA concentration and purity were determined using NanoDrop 2000 UV-vis spectrophotometer (Thermo Scientific, Wilmington, United States). The hypervariable V3-V4 region of the 16S rRNA gene with a length of ~468 bp was targeted for sequencing. PCR amplification was performed with gene-specific primers 338 F (5′-ACTCCTACGGGAGGCAGCAG-3′) and 806 R (5′-GGACTACHVGGGTWTCTAAT-3′) under the following conditions: initial denaturation at 95°C for 3 min, followed by 27 cycles of denaturing at 95°C for 30 s, annealing at 55°C for 30 s, extension at 72°C for 45 s, single extension at 72°C for 10 min, and end at 4°C.

The PCR mixtures contain 5 × TransStartFastPfu buffer 4 μl, 2.5 mM dNTPs 2 μl, forward primer (5 μM) 0.8 μl, reverse primer (5 μM) 0.8 μl, TransStartFastPfu DNA Polymerase 0.4 μl, template DNA 10 ng, and finally ddH_2_O up to 20 μl. PCR reactions were performed in triplicate. The PCR product was extracted from a 2% agarose gel, purified using the AxyPrep DNA Gel Extraction Kit (Axygen Biosciences, Union City, CA, United States) according to the manufacturer's instructions, and quantified using the Quantus™ Fluorometer (Promega, United States). Purified amplicons were pooled in equimolar amounts and paired-end sequenced on an Illumina MiSeq PE300 platform (Illumina, San Diego, United States) according to the standard protocols by Majorbio Bio-Pharm Technology Co. Ltd. (Shanghai, China). All obtained raw sequence datasets have been deposited into the National Center for Biotechnology Information (NCBI) Sequence Read Archive (SRA) with the accession number PRJNA799073.

The raw 16S rRNA gene sequencing reads were demultiplexed, quality-filtered by the FastP version 0.20.0 (Chen et al., 2018) and merged by the FLASH version 1.2.7 (Magoc and Salzberg, 2011) with the following criteria: the 300 bp reads were truncated at any site receiving an average quality score of <20 over a 50 bp sliding window, and the truncated reads shorter than 50 bp were discarded; reads containing ambiguous characters were also discarded; only overlapping sequences longer than 10 bp were assembled according to their overlapped sequence. The maximum mismatch ratio of the overlap region is 0.2. Reads that could not be assembled were discarded; samples were distinguished according to the barcode and the primers, and the sequence direction was adjusted for exact barcode matching and a 2-nucleotide mismatch in primer matching. Operational taxonomic units (OTUs) with a 97% similarity cutoff (Schloss et al., 2009) were clustered using the UPARSE version 7.1, and chimeric sequences were identified and removed. The taxonomy of each OTU representative sequence was analyzed by RDP Classifier version 2.2 (Wang et al., 2007) against the 16S rRNA database (Silva version 138) using a confidence threshold of 0.7.

### Statistical analysis

Reproductive performance, including HEL, FE, and CN, was calculated using the SAS 9.4 software (Shen et al., 2014). Alpha diversity was calculated using the Mothur (Schloss et al., 2009). Venn diagrams and rank abundance distribution curves were performed using Mothur. Linear discriminate analysis effect size (LEfSe) was used to identify the bacteria enriched (Segata et al., 2011). The pair-wise phylogenetic distance was measured by weighted UniFrac (Lozupone et al., 2006) to compare community compositions across samples. Principal component analysis (PCA) was used to compress dimensionality into 2D principal coordinate analysis plots (Vazquez-Baeza et al., 2013), enabling visualization of sample relationships. PICRUSt was used to explore the functional composition of that bacterial community that the data might convey (Langille et al., 2013). The visualization of conventional results was achieved by Origin 2023. The co-occurrence network is implemented through the Gephi 0.10 software.

## Results

### Comparisons of reproductive performances in *S. pullorum*-negative hens and *S. pullorum*-positive hens

**Figure 1:**
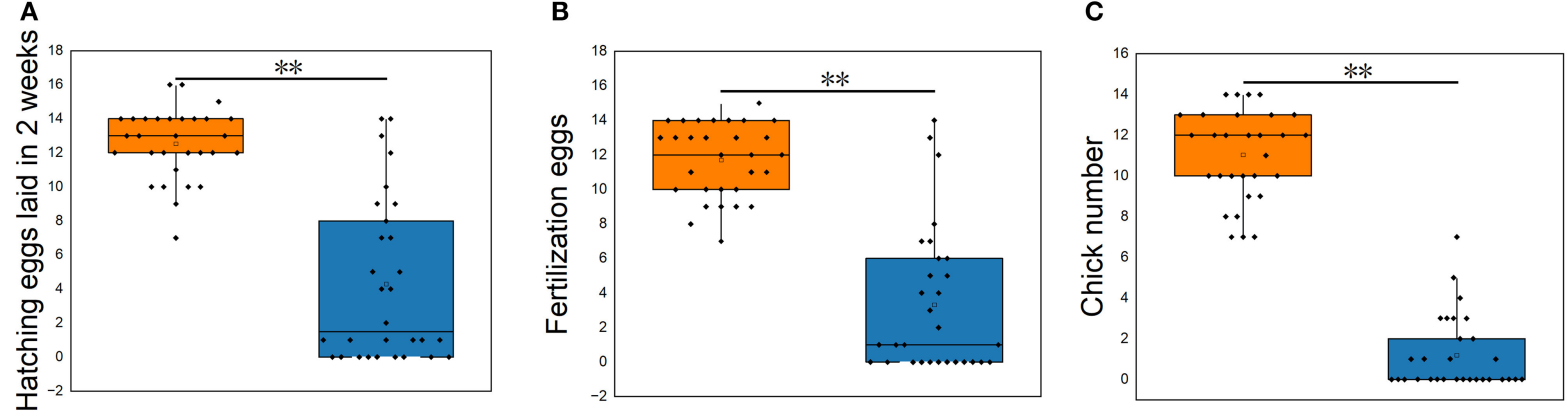
**(A–C)** Comparisons of reproductive performances in *S. pullorum*-negative hens and *S. pullorum*-positive hens. PN, *S. pullorum*-negative group; PP, *S. pullorum*-positive group.

The reproductive performance showed that the average hatching eggs laid in 2 weeks (HEL), average fertilization eggs (FE), and average chick number (CN) of *S. pullorum*-negative hens were higher than that of *S. pullorum*-positive hens (*p* < 0.01, Figure 1). The average HEL of *S. pullorum*-negative hens was 12.5, which was 0.5 more than that of *S. pullorum*-positive hens. The average FE of *S. pullorum*-negative hens was 11.7, which was 8.4 times more than that of *S. pullorum*-positive hens. The average CN of *S. pullorum*-negative hens was 11.0, which was 9.8 times more than that of *S. pullorum*-positive hens.

### Morphological observation of the reproductive tract and intestinal tract

**Figure 2:**
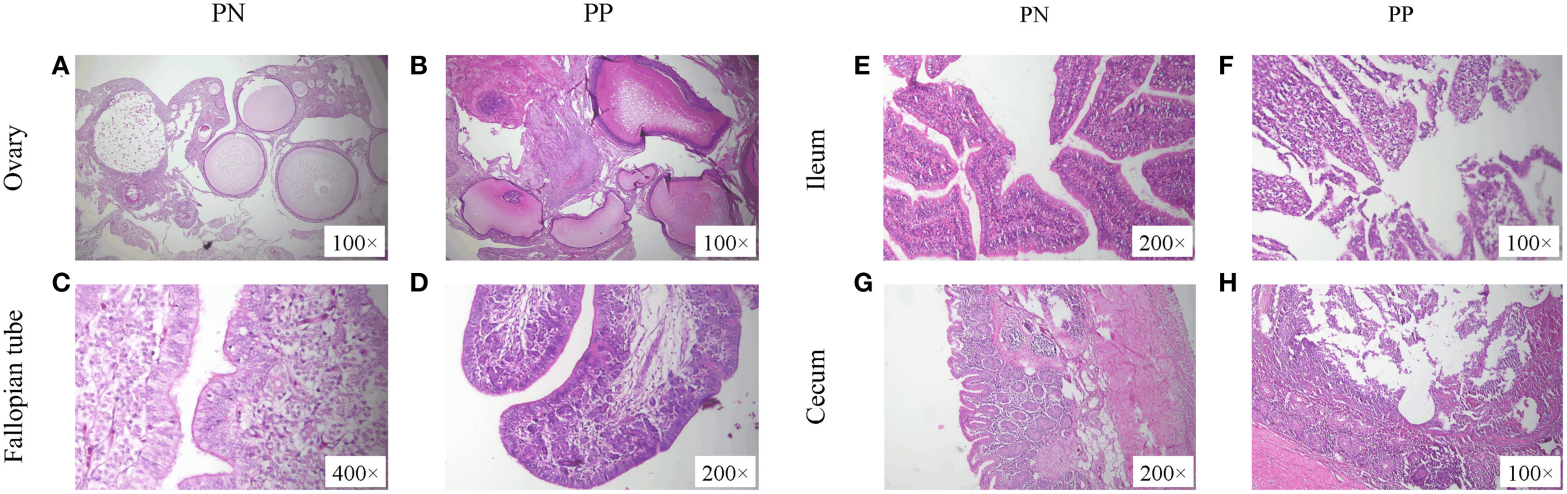
**(A–H)** Morphological observation of ovaries, fallopian tubes, ileum, and cecum of PN and PP. PN, *S. pullorum*-negative group; PP, *S. pullorum*-positive group.

Morphological observations showed that the presence of deformed follicles, follicular dysplasia, or even necrosis occurred in the *S. pullorum*-positive hens (Figures 2A, B). Moreover, in the *S. pullorum*-positive hens, epithelial cells of the oviductal mucosa consisted mostly of ciliated cells, with few secretory cells and an underdeveloped lamina propria (Figures 2C, D). Histomorphological observations of the ileum and cecum in PP revealed that the intestinal epithelial integrity was low and there was severe intestinal epithelial damage. Severe detachment of mucosal epithelial cells and exposure of the lamina propria in the intestinal lumen were mainly observed (Figures 2E, H).

### Bacterial community structure and prediction functions of *S. pullorum*-negative hens and *S. pullorum*-positive hens

**Figure 3:**
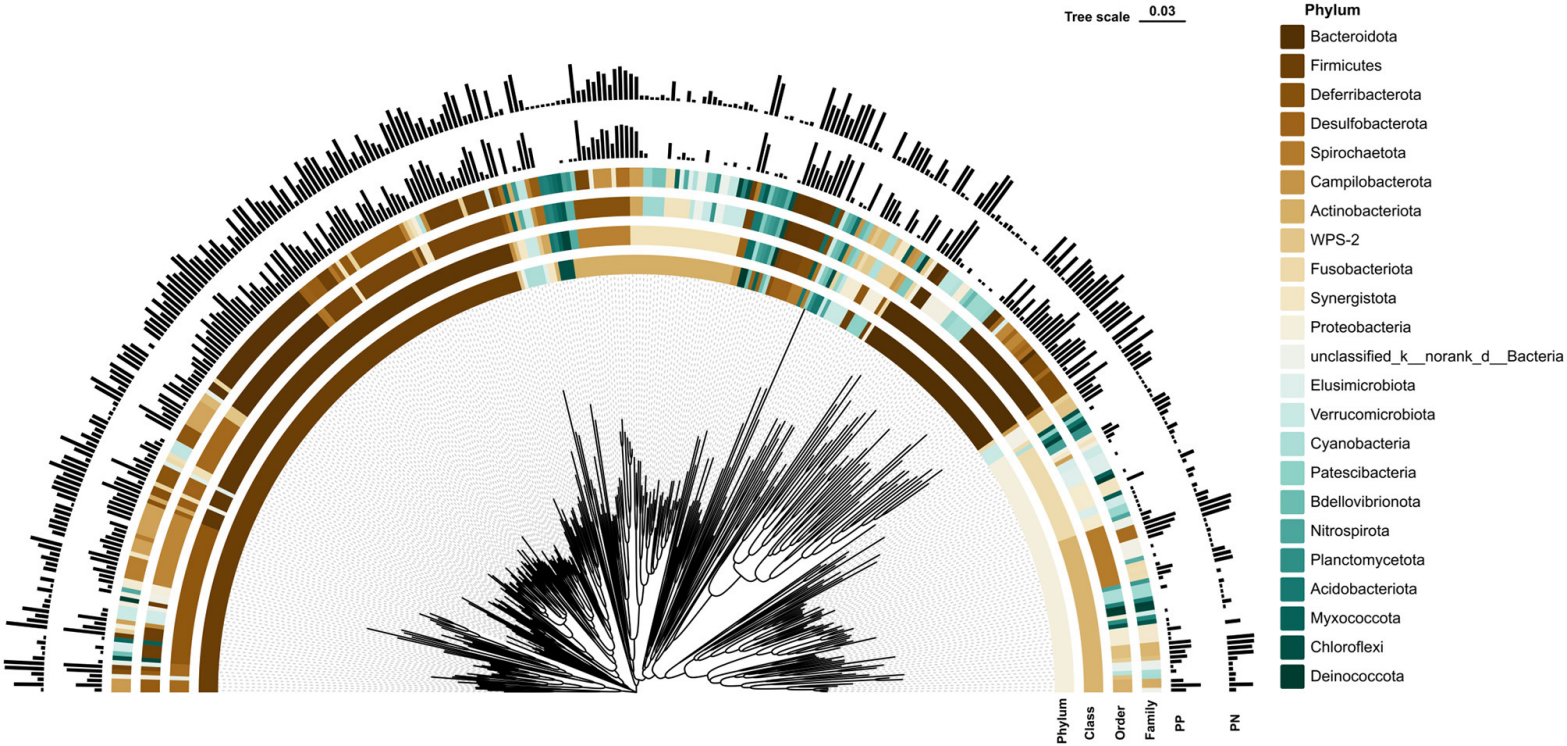
Phylogenetic tree of cecal microbiota and its microbial community composition in PN and PP. PN, *S. pullorum*-negative group; PP, *S. pullorum*-positive group.

**Figure 4:**
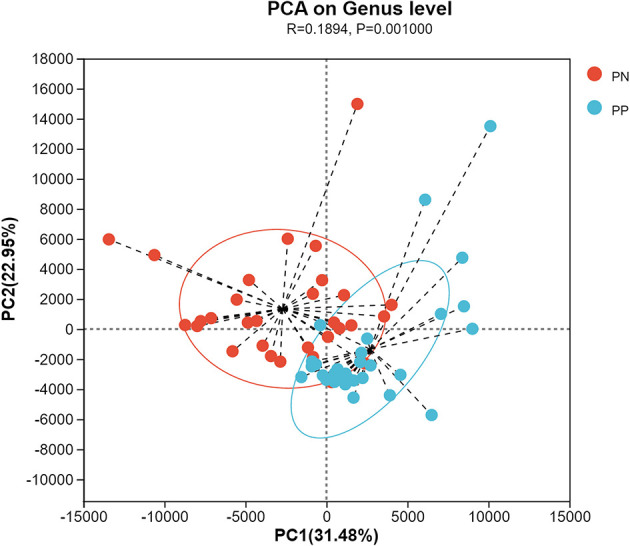
Principal component analysis (PCA) at genus level between PN and PP. PN, *S. pullorum*-negative group; PP, *S. pullorum*-positive group.

**Figure 5:**
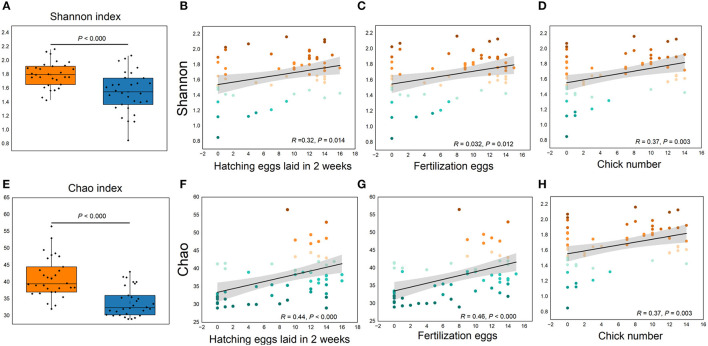
**(A–H)** Cecum microbial alpha diversity and its relationships with reproductive performance. **(A, E)**, Shannon and Chao indexes, respectively. **(B, D, F, H)** Correlations between reproductive performance with Shannon and Chao indexes, respectively.

More than 4 million sequences were obtained from all samples, and there were 16,888 high-quality sequences per sample. The average sequence length was 417 bp. Microbial composition analysis showed that, at the phylum level, the two most dominant phyla were *Firmicutes* and *Bacteroidetes*, which comprised 72.6% of the total sequences in PN and 71.6% of the total sequences in PP, respectively (Figure 3). At the genus level, a total of 357 genera were identified from all samples, and the enrichment of these genera in the PN and PP had a large variation (Figure 3). The two most dominant genera were *Bacteroides* and *Rikenellaceae_RC9_gut_group* belonging to the phylum *Bacteroidota* and comprised 25.70% of the total sequences in PN and 24.42% of the total sequences in PP, respectively (Figure 3). A total of 1,880 OTUs were identified from all samples, and the VENN diagram showed the unique OTUs and genera of the two groups and the shared OTUs and genera (Supplementary Figure S1). In addition, principal component analysis (PCA) at the genus level revealed a significant separation between the samples of PN and PP, indicating a large difference in the cecum microbiota of these two groups (Figure 4). The diversity and richness of the cecum microbiota were significantly lower in the PP group than in the PN group (Figures 5A, E).

**Figure 6:**
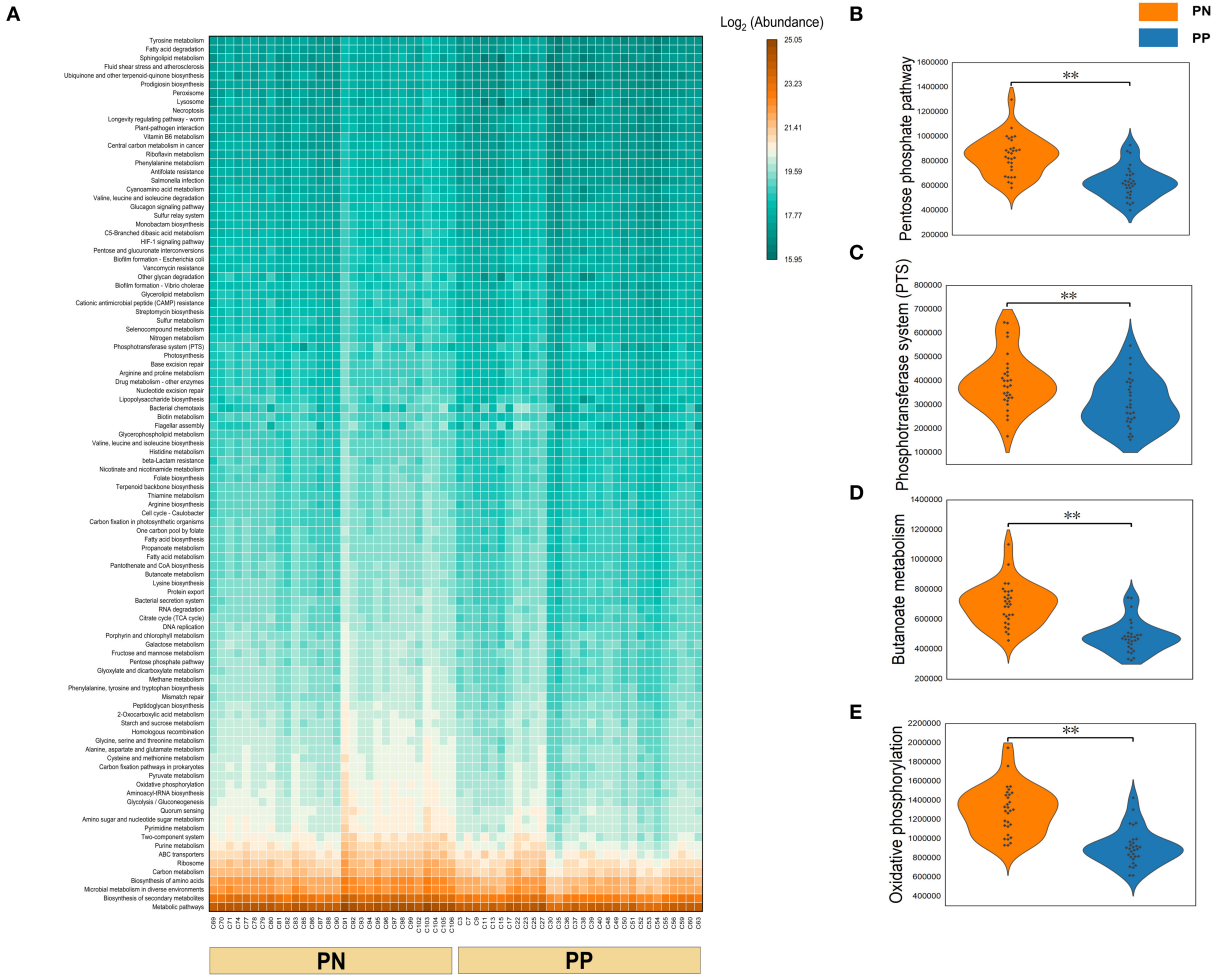
**(A–E)** Functional composition of microbiota and their differences between two groups. Microbial functional composition. **(A)** Heatmap of the functional composition (top 100) of microbiota. **(B–E)** Comparison of abundance of phosphotransferase system (PTS) and pentose phosphate pathway, butanoate metabolism, and oxidative phosphorylation.

A Phylogenetic Investigation of Communities by Reconstruction of Unobserved States (PICRUSt) analysis was performed to investigate the functional properties of microbiota. Using the KEGG pathway annotation information, we found that among the major microbial functions (top 100), the PP cecum microbial functions were generally weaker than the PN group (Figure 6A), where the phosphotransferase system (PTS), pentose phosphate pathway, butanoate metabolism, and oxidative phosphorylation were significantly less abundant in PP than PN (Figures 6B–E).

### Correlations of reproductive performances with the gut microbiota

**Figure 7:**
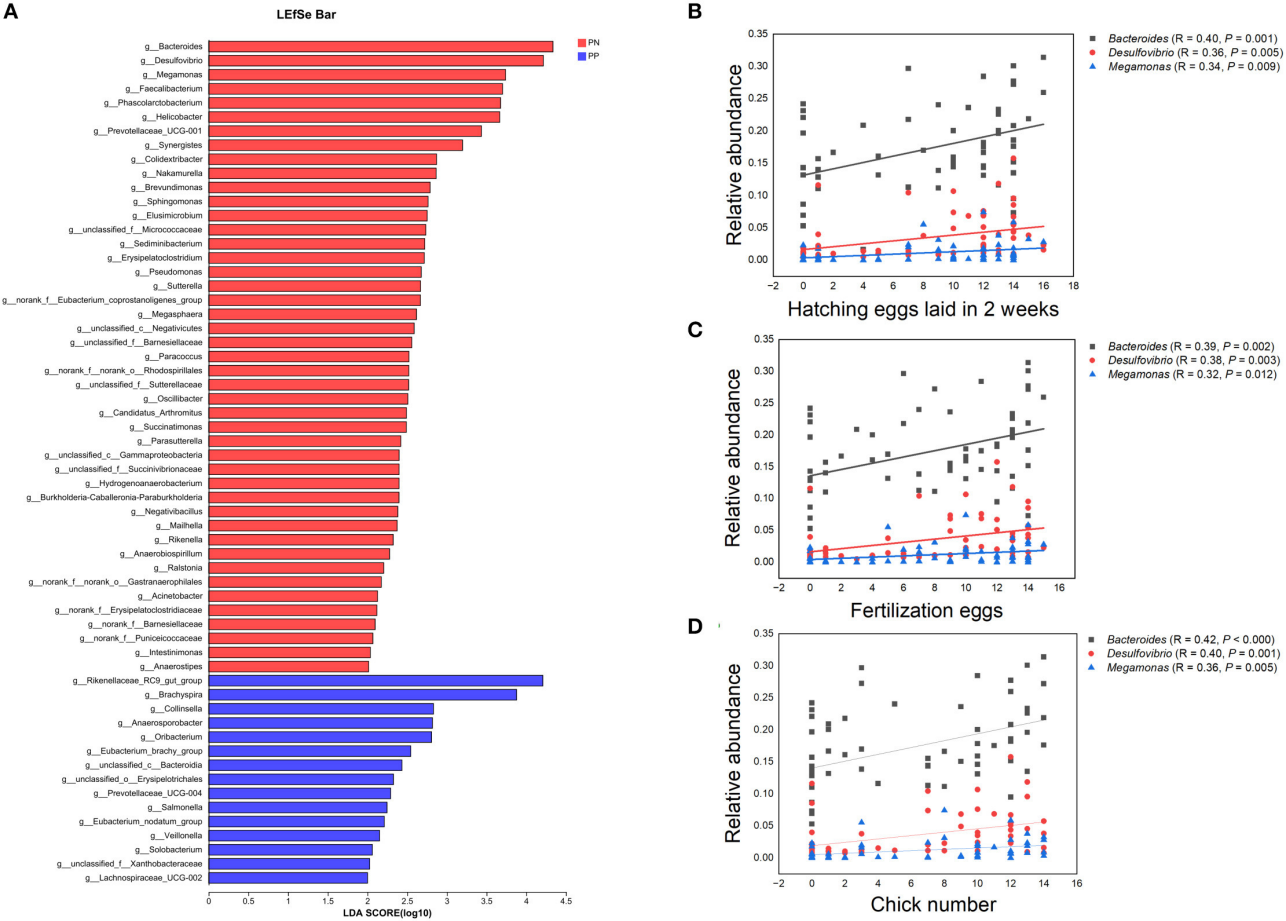
**(A–D)** Identification of differential microbes and the associations between microbial abundance and reproductive performance. **(A)** Linear discriminant analysis (LDA) effect size (LEfSe) analysis. **(B–D)** Correlation analysis microbial abundance and reproductive performance.PN, *S. pullorum*-negative group, PP, *S. pullorum*-positive group.

It is noteworthy that the alpha diversity of the cecum microbiota was positively correlated with the reproductive performance (HEL, FE, and CN) of hens (*p* < 0.01, Figures 5B–D, F–H). To further investigate the microbial differences between the PP and PN groups, the Wilcoxon rank-sum test was used, and it was found that there were 68 significantly different genera between these two groups (*p* < 0.05, Supplementary Table S2). LEfSe was used to further determine the taxa that most likely explain the differences between PN and PP samples. A total of 65 genera were found to be potential biomarkers between PN and PP. There was a significant enrichment of 50 genera (including *Bacteroides, Desulfovibrio*, and *Megamonas*) in PN (Figure 7 A). Additionally, enrichment of 15 genera, including *Salmonella*, was found in PP (Figure 7A). Notably, we found significant positive correlations between *Bacteroides, Desulfovibrio*, and *Megamonas*, with reproductive performance (HEL, FE, and CN, Figures 7B–D).

### Analysis of the co-occurrence network of microorganisms between *S. pullorum*-negative hens and *S. pullorum*-positive hens

**Figure 8:**
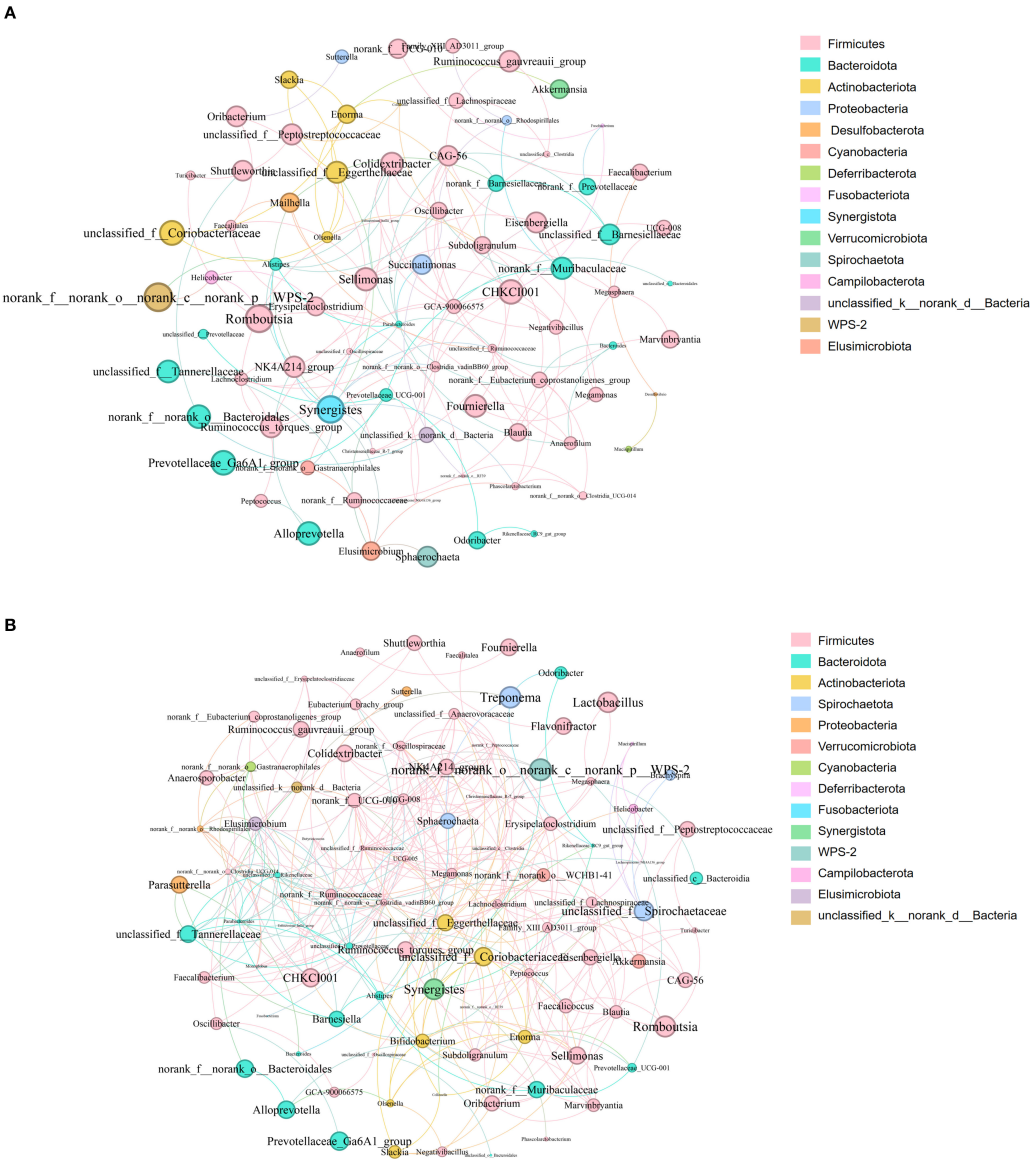
**(A, B)** Microbial co-occurrence network analysis at the genus level. **(A)** was PN, **(B)** was PP. Node size indicates relative abundance. Red edges indicate significant positive correlation (*P* < 0.05), and green edges indicate significant negative correlation (*p* < 0.05). PN, *S. pullorum*-negative group; PP, *S. pullorum*-positive group.

Analysis of the co-occurrence network of microorganisms at the genus level found that in the PP and PN groups, the genus *Firmicutes* is the core microbe in the co-occurrence network and has extensive connections with microbes from other phyla (Figure 8). In addition, compared with the control group, the association between members of the *Firmicutes* phylum in the PP group seems to be closer (Figure 8B).

## Discussion

Pullorum is a disease caused by *S. pullorum* through both horizontal and vertical transmission. It is known that a percentage of birds that survive clinical disease when they are infected as young chicks may show few signs of infection but may become carriers. Intestinal carriage of *S. pullorum* in the poultry host does not cause substantive gastrointestinal disease and is asymptomatic. Therefore, it is of great significance to identify gut microbiota related to pullorum disease in chickens. This will lead to the development of novel prevention and control strategies for *S. pullorum* infection in poultry production.

Among the different diseases occurring in poultry, those caused by the genus *Salmonella* are the most common, leading to serious economic losses to the poultry industry in terms of mortality, reduced growth, and loss of egg production (Shivaprasad, 2000; Wigley et al., 2001). One aim of the current study was to investigate and characterize the effect that *S. pullorum* infection had on reproductive performances. The results illustrated that *S. pullorum* infection in chickens reduced their reproductive performances and altered the gut microbial composition, resulting in variations in the microbial metabolic pathways and functions. Previous studies have reached the same conclusion (Ding et al., 2021). This study illustrated that pullorum disease reduced reproductive performance including HEL, FE, and CN. Pullorum disease is manifested by decreased egg production, fertility, and hatchability in hens. Shivaprasad HL showed that regressing ovarian follicles can be found in the ovaries of chickens and microscopic lesions in adults include fibrinosuppurative to pyogranulomatous inflammation of ovarian follicles characterized by necrosis. Caeca may contain necrotic caseous debris within the lumen and necrosis of the mucosa with the infiltration of heterophils into the lamina propria (Shivaprasad, 2000). The other aim of this study was to investigate the different bacterial communities between *S. pullorum*-negative hens and *S. pullorum*-positive hens and their microbial functions. The availability of high-throughput sequencing will shortly enable the sequencing of whole bacterial populations, giving us a more comprehensive view of bacterial evolution among related bacterial species (Barrow and Freitas Neto, 2011). Shen et al. studied the dissemination pattern of *S. pullorum* in different organs and at different time points. It showed that the cecum carried *S. pullorum* throughout the experiment duration, while the small intestine did not carry *S. pullorum* during the last few days of the experiment (Shen et al., 2022). Therefore, this study examined DNA sequence data and the bacterial community structure of *S. pullorum*-negative hens and *S. pullorum*-positive hens in the cecum. It obtained a large number of effective sequences. The two most dominant phyla were *Firmicutes* and Bacteroidota. The similar results were obtained in previous studies (Wei et al., 2013; Khan et al., 2020; Rychlik, 2020). The two most dominant genera were *Bacteroides* and *Rikenellaceae_RC9_gut_group*, which belong to the phylum *Bacteroidota* comprised 34.1% of the total sequences in PN and 16.2% of the total sequences in PP, respectively. Ding *et al*. investigated that the dominant phyla were *Firmicutes* (65.5% in group N and 62.1% in group P), *Fusobacteria* (16.3% in group N and 18.7% in group P), and *Proteobacteria* (9.37% in group N and 9.95% in group P); the preponderant genera were *Lactobacillus, Fusobacterium, Peptoclostridium*, and *Gallibacterium* (Ding et al., 2021). The reasons that lead to different results in similar studies are complicated. Many factors can cause different microbiome compositions (e.g., breeds, age, gender, nutritional level, and sample selection). It is interesting that although significant enrichment of *Salmonella* was found in PN, there was a low abundance of *Salmonella* in PP. The basis of host specificity in salmonellosis continues to elude scientific explanation (Barrow et al., 2012). The outcome of infection is the combined effect of the microbial gene set and the host's genetic background. After an intestinal infection, where are the sites of *Salmonella* serovar Pullorum persistence in convalescent birds? Wigley *et al*. showed that *Salmonella* serovar Pullorum evades the immune response by surviving intracellularly within macrophages, which would be required by *Salmonella* serovar Pullorum to persist in both the spleen and the reproductive tract (Wigley et al., 2001). *Salmonella* serovar Pullorum localizes in the reproductive tract of chickens and, as a consequence, may be transmitted vertically to chicks by transovarian transmission of the bacteria into developing hatching eggs (Wigley et al., 2001). The correlation analysis between reproductive performances (HEL, FEN, and CN) and gut microbiota was therefore significant.

After a systemic disease, there may be negative effects on immunity, such as the balance of gut microbes, which could be upset, and the ability of harmful bacteria to secrete toxins, which could be increased. In this study, the ileum and cecum in PP revealed low intestinal epithelial integrity, and this should cause negative effects in the absorption and digestion of feed nutrients. Intestinal integrity is essential to prevent animal bacterial diseases (Citi, 2018). A stable and healthier intestinal state can inhibit pathogenic bacteria and reduce the production of toxins to ensure better capacity for digestion and absorption in hens (Jackman et al., 2020; Papadopoulos et al., 2022; Cui et al., 2023). The gut microbiota is widely perceived as being closely related to gut health and the growth performance of the host (Wang et al., 2016). The results elaborated that the abundances of 29, 32, and 39 genera were separately positively correlated with HEL, FEN, and CN. Meanwhile, the abundances of 7, 6, and 2 genera were separately negatively correlated with HEL, FEN, and CN.

The 50 genera, which were significant enrichments for *S. pullorum*-negative hens, were used to make PICRUSt1 function prediction analyses in all samples. The most important functions and metabolic pathways of the above different potential biomarkers were amino acid transport and metabolism and amino acid metabolism, respectively. Among these 50 genera, previous studies showed that *Bacteroides* and *Megamonas* could produce short-chain fatty acids (SCFA) by fermenting carbohydrates to provide energy for the gut and promote the growth performance of animals (Hooper et al., 2002; Shimizu et al., 2019; Ni et al., 2021; Zhu et al., 2021). *Desulfovibrio* consumes free hydrogen for the reduction of sulfate, thus contributing to the removal of free hydrogen formed during anaerobic fermentation in the gut environment (Rychlik, 2020). *Faecalibacterium* as a probiotic candidate has shown promising results toward enhancing food safety and gut health (Khan et al., 2020). The major source energies of *Suterrella* and *Parasutterella* originate from protein, amino acid, and fatty acid metabolism (Line et al., 2010; Polansky et al., 2015). Rychlik showed that *Megasphaera* and *Phascolarctobacterium* were capable of butyrate production (Rychlik, 2020). Zhang indicated that high body weight chickens contained *Sphingomonas* more abundantly (*p* < 0.05) (Zhang et al., 2021). In-feed supplementations of probiotics strengthen the gut microbiota for improved host performance and colonization resistance to gut pathogens such as *Salmonella* and *Campylobacter*. The mechanisms of action of prebiotics and probiotics come through the production of organic acids, the activation of the host immune system, and the production of antimicrobial agents. Many probiotic preparations contain high numbers of lactobacilli that normally produce large quantities of volatile fatty acids such as formic acid. The incorporation of these into feed has been shown to inhibit gut colonization by zoonotic serovars of *Salmonella* (Barrow and Freitas Neto, 2011). Mixed probiotics effectively reduced the mortality of pullorosis in chicks, promoted growth performance, regulated the balance of the intestinal flora, improved immune function, resisted pullorosis disease, completely prevented chicks from pullorosis after infection, and reduced economic loss in the poultry industry (Chen et al., 2020). The probiotics reported above could be in culture, but most of these were poor or unclear, and most studies are empirical in nature.

Many studies have revealed the importance of probiotics in the context of infectious diseases, including pullorum. Zhou *et al*. studied the effect of a selected yeast fraction (*Safmannan*, SYF) on the prevention of pullorum disease in commercial breeder chickens and demonstrated that SYF supplementation could significantly decrease SP and SG infection rates and improve the body weight of birds challenged with *S. pullorum* (Zhou et al., 2020). Mon *et al*. examined the three-way interaction that occurred between host metabolites, resident gut microbiota, and *Salmonella* following inoculation of *Salmonella enteritidis* in 2-week-old layer chicks. It showed that there was differential regulation in many of the metabolites in association with *Salmonella enteritidis* colonization in chickens; perturbation in metabolic pathways related to arginine and proline metabolism as well as the TCA cycle was most prominently detected (Mon et al., 2020). Alrubaye *et al*. learned that the microbial metabolite deoxycholic acid shapes microbiota against *Campylobacter jejuni* chicken colonization and suggested that there was a bidirectional interaction between microbiota and microbial metabolites (Alrubaye et al., 2019). In this study, most of the genera we obtained by 16S rRNA sequencing were uncultured. The use of competitive exclusion gut flora preparations has the same protective effect as the normal flora in animal intestines. There is the possibility of a very intimate interaction between host bacteria and pathogens in the cecum (Barrow et al., 2012), and one area for future *Salmonella* control exploration is the development of probiotic organisms that have a rational basis for protection. Utilization of the information generated in this study should improve the efficacy of surveillance and biological interventions, both for intestinal carriage.

## Conclusion

Pullorum disease reduced reproductive performance. Abnormal morphology of the ovaries and fallopian tubes and low integrity of epithelial tissue in the ileum and cecum were found in PP. Pullorum disease reduced the cecal microbial alpha diversity and relative abundance of *Bacteroides, Desulfovibrio*, and *Megamonas*, which were positively correlated with reproductive performance. Diminished phosphotransferase systems (PTS) and pentose phosphate pathways, butanoate metabolism, and oxidative phosphorylation were also found in PP. Taken together, this study clarified the morphological characteristics of the reproductive tract and intestines of hens infected with *S. pullorum* and preliminarily explored the potential association between cecal microbiota and reproductive performance in hens.

## Data availability statement

The datasets presented in this study can be found in online repositories. The names of the repository/repositories and accession number(s) can be found below: https://www.ncbi.nlm.nih.gov/, PRJNA799073.

## Ethics statement

The animal study was reviewed and approved by the Ethics and Animal Welfare Committee of Shanghai Academy of Agricultural Sciences (No. SAASPZ0521009). Written informed consent was obtained from the owners for the participation of their animals in this study.

## Author contributions

QN and QH conceived and designed the experiments. CG, ZZ, and KY raised the experimental animals. XW, CC, XQ, and QN participated in the sample collection. XQ and QN participated in the data analysis. QN wrote the article. QH, XW, CC, XQ, CG, KY, and ZZ assisted with experiments and provided advice on manuscript content. All authors read and approved the final manuscript.

## Supplementary material

Supplementary Figure S1 Venn diagrams of OTUs **(A)** and genera **(B)** between PN and PP, respectively. PN, *S. pullorum*-negative group; PP, *S. pullorum*-positive group.

Supplementary table S1 Composition of experimental diet.

Supplementary table S2 The microbes with significant differences between PN and PP.
