# Supplementary figures and images for: Identification of the gut microbiota affecting Salmonella pullorum and their relationship with reproductive performance in hens
Source: Front Microbiol. 2023 Jul 27;14:1216542. doi: 10.3389/fmicb.2023.1216542 (PMC10413576; doi:10.3389/fmicb.2023.1216542)

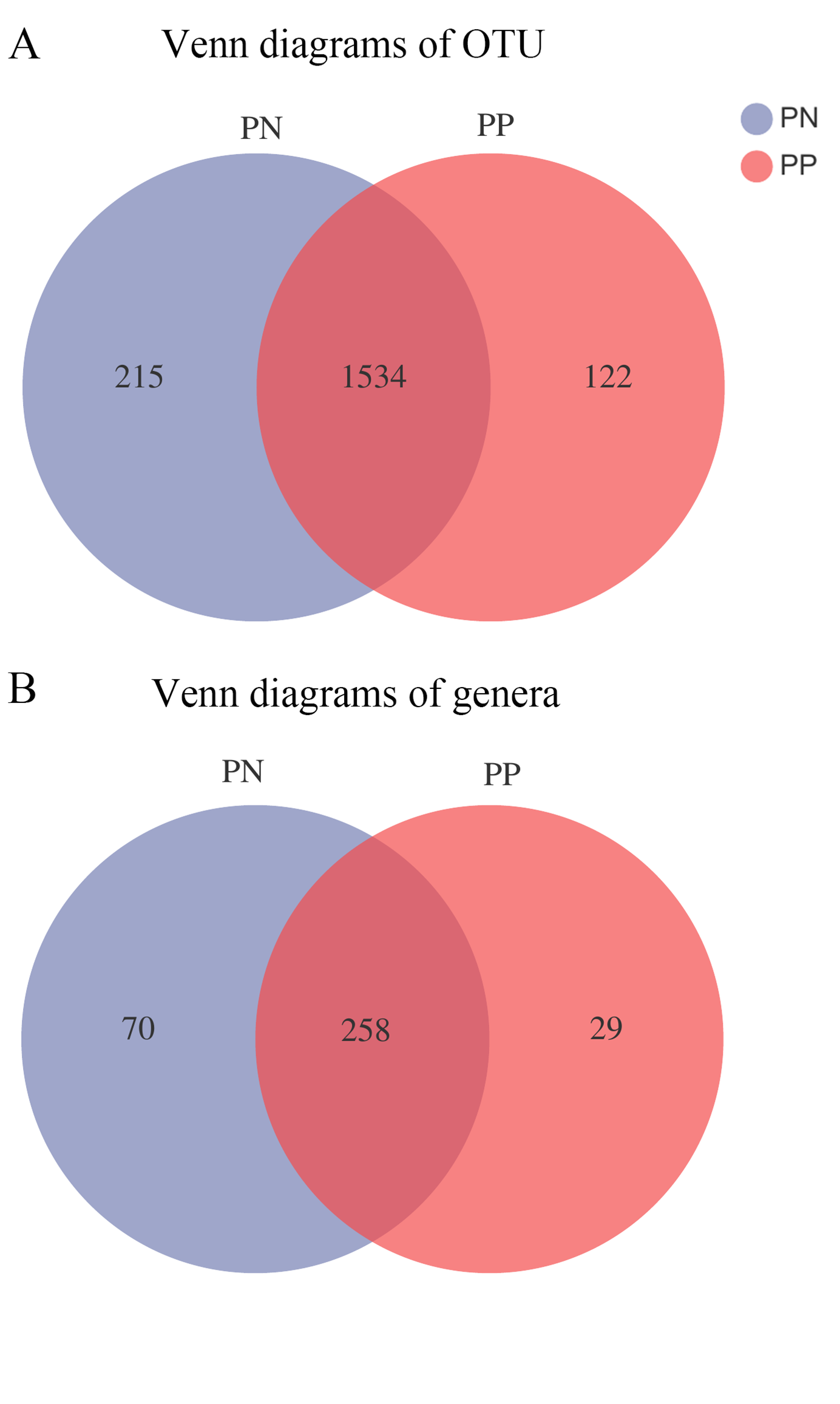

Supplement: Supplementary Figure S1 — Venn diagrams of OTUs (A) and genera (B) between PN and PP, respectively. PN, S. pullorum-negative group; PP, S. pullorum-positive group. [file Image_1.TIF]
