# Supplementary material for: Identification of the gut microbiota affecting Salmonella pullorum and their relationship with reproductive performance in hens
Source: Front Microbiol. 2023 Jul 27;14:1216542. doi: 10.3389/fmicb.2023.1216542 (PMC10413576; doi:10.3389/fmicb.2023.1216542)
Supplement: Supplementary table S1 — Composition of experimental diet. [file Table_1.DOCX]

**Table S1** Composition of experimental diet.

| Diet composition | Content (%) |
| --- | --- |
| Corn | 63 |
| Soybean meal | 24 |
| Wheat Bran | 1.5 |
| Stone powder | 9 |
| Premix^1^ | 2.5 |

^1^Premix: VA (IU/kg): 15400; VD3 (IU/kg): 3300; VE (IU/kg): 33; VK3 (mg/kg): 2.2; VB1 (mg/kg): 2.2; VB2 (mg/kg): 9.9; VB6 (mg/kg): 5.5; VB12 (mg/kg): 0.013; Niacin (mg/kg): 44; Pantothenic acid (mg/kg): 13.2; Folic acid (mg/kg): 1.65; Choline (mg/kg): 440; Biotin (mg/kg): 0.22; Antioxidant (mg/kg): 120; Manganese (mg/kg): 120; Zinc (mg/kg):110; Iron (mg/kg): 40; Iodine (mg/kg): 1.1; Copper (mg/kg): 8; Selenium (mg/kg): 0.3.
