# Supplementary material for: Identification of the gut microbiota affecting Salmonella pullorum and their relationship with reproductive performance in hens
Source: Front Microbiol. 2023 Jul 27;14:1216542. doi: 10.3389/fmicb.2023.1216542 (PMC10413576; doi:10.3389/fmicb.2023.1216542)
Supplement: Supplementary table S2 — The microbes with significant differences between PN and PP. [file Table_2.DOCX]

**Table S2** The microbes with significant differences between PN and PP.

| Genera | PN | | PP | | p value |
| --- | --- | --- | --- | --- | --- |
|  | Mean(%) | SD | Mean(%) | SD |  |
| Bacteroides | 19.2800 | 5.8730 | 15.3300 | 7.0620 | 0.030 |
| Rikenellaceae_RC9_gut_group | 6.5370 | 2.6160 | 9.8730 | 5.8790 | 0.022 |
| Desulfovibrio | 5.1190 | 3.7350 | 1.9360 | 2.4000 | 0.000 |
| Helicobacter | 2.7100 | 2.9500 | 1.7260 | 2.5840 | 0.048 |
| Faecalibacterium | 2.4930 | 1.9260 | 1.5080 | 1.1900 | 0.018 |
| Brachyspira | 1.3180 | 1.9420 | 2.6650 | 5.1090 | 0.040 |
| Phascolarctobacterium | 2.2710 | 1.1620 | 1.3270 | 0.8888 | 0.002 |
| Prevotellaceae_UCG-001 | 1.6090 | 1.4930 | 1.0760 | 0.9290 | 0.041 |
| Megamonas | 1.7020 | 1.7560 | 0.6064 | 1.1350 | 0.000 |
| Synergistes | 1.0960 | 0.8324 | 0.7726 | 0.6648 | 0.047 |
| Sutterella | 0.3842 | 0.2062 | 0.3391 | 0.3680 | 0.015 |
| Colidextribacter | 0.3123 | 0.1629 | 0.1663 | 0.0916 | 0.000 |
| Negativibacillus | 0.2633 | 0.1002 | 0.2145 | 0.1427 | 0.035 |
| Oribacterium | 0.1762 | 0.0703 | 0.2996 | 0.1880 | 0.000 |
| Erysipelatoclostridium | 0.2878 | 0.2276 | 0.1806 | 0.1245 | 0.009 |
| Elusimicrobium | 0.2695 | 0.2645 | 0.1472 | 0.1798 | 0.018 |
| Parasutterella | 0.2338 | 0.0877 | 0.1786 | 0.1150 | 0.038 |
| Collinsella | 0.1261 | 0.0799 | 0.2481 | 0.2446 | 0.007 |
| norank_f__Eubacterium_coprostanoligenes_group | 0.2031 | 0.1026 | 0.1074 | 0.0860 | 0.000 |
| Oscillibacter | 0.1743 | 0.0704 | 0.1111 | 0.0556 | 0.001 |
| norank_f__norank_o__Rhodospirillales | 0.1313 | 0.0916 | 0.0656 | 0.0652 | 0.001 |
| Megasphaera | 0.1375 | 0.1445 | 0.0547 | 0.0915 | 0.009 |
| Eubacterium_brachy_group | 0.0537 | 0.0485 | 0.1234 | 0.1321 | 0.002 |
| Anaerosporobacter | 0.0085 | 0.0139 | 0.1492 | 0.3632 | 0.001 |
| unclassified_f__Barnesiellaceae | 0.1088 | 0.1033 | 0.0391 | 0.0499 | 0.000 |
| unclassified_f__Sutterellaceae | 0.0966 | 0.0578 | 0.0391 | 0.0316 | 0.000 |
| norank_f__norank_o__Gastranaerophilales | 0.0806 | 0.0725 | 0.0548 | 0.0662 | 0.039 |
| norank_f__Barnesiellaceae | 0.0626 | 0.0576 | 0.0412 | 0.0571 | 0.014 |
| Succinatimonas | 0.0760 | 0.0436 | 0.0195 | 0.0171 | 0.000 |
| Mailhella | 0.0657 | 0.0415 | 0.0215 | 0.0152 | 0.000 |
| unclassified_f__Succinivibrionaceae | 0.0598 | 0.2136 | 0.0196 | 0.0442 | 0.028 |
| Candidatus_Arthromitus | 0.0663 | 0.1573 | 0.0024 | 0.0040 | 0.000 |
| Eubacterium_nodatum_group | 0.0198 | 0.0218 | 0.0471 | 0.0601 | 0.045 |
| unclassified_c__Gammaproteobacteria | 0.0540 | 0.1534 | 0.0064 | 0.0220 | 0.024 |
| unclassified_c__Bacteroidia | 0.0000 | 0.0000 | 0.0569 | 0.1164 | 0.000 |
| norank_f__Victivallaceae | 0.0271 | 0.0154 | 0.0199 | 0.0184 | 0.041 |
| Anaerobiospirillum | 0.0383 | 0.0642 | 0.0040 | 0.0064 | 0.003 |
| Tuzzerella | 0.0233 | 0.0167 | 0.0169 | 0.0171 | 0.048 |
| Lachnospiraceae_UCG-002 | 0.0133 | 0.0083 | 0.0252 | 0.0214 | 0.036 |
| Bilophila | 0.0203 | 0.0153 | 0.0153 | 0.0255 | 0.025 |
| Solobacterium | 0.0066 | 0.0169 | 0.0249 | 0.0659 | 0.041 |
| Family_XIII_UCG-001 | 0.0145 | 0.0107 | 0.0088 | 0.0069 | 0.027 |
| unclassified_f__Desulfovibrionaceae | 0.0111 | 0.0088 | 0.0066 | 0.0049 | 0.048 |
| ASF356 | 0.0130 | 0.0318 | 0.0016 | 0.0039 | 0.013 |
| norank_f__Puniceicoccaceae | 0.0109 | 0.0143 | 0.0025 | 0.0028 | 0.000 |
| Rhodococcus | 0.0086 | 0.0100 | 0.0035 | 0.0046 | 0.010 |
| Intestinimonas | 0.0072 | 0.0061 | 0.0032 | 0.0047 | 0.000 |
| Ralstonia | 0.0075 | 0.0070 | 0.0018 | 0.0034 | 0.000 |
| unclassified_o__Erysipelotrichales | 0.0017 | 0.0022 | 0.0074 | 0.0368 | 0.005 |
| unclassified_f__Xanthobacteraceae | 0.0025 | 0.0058 | 0.0064 | 0.0075 | 0.005 |
| Anaerostipes | 0.0068 | 0.0157 | 0.0014 | 0.0032 | 0.008 |
| norank_f__Erysipelatoclostridiaceae | 0.0064 | 0.0104 | 0.0016 | 0.0026 | 0.009 |
| Hydrogenoanaerobacterium | 0.0035 | 0.0031 | 0.0020 | 0.0024 | 0.043 |
| Acinetobacter | 0.0031 | 0.0031 | 0.0019 | 0.0032 | 0.024 |
| Veillonella | 0.0003 | 0.0009 | 0.0032 | 0.0077 | 0.010 |
| unclassified_o__Rhodospirillales | 0.0025 | 0.0050 | 0.0005 | 0.0019 | 0.015 |
| Rikenella | 0.0017 | 0.0026 | 0.0007 | 0.0022 | 0.008 |
| Salmonella | 0.0003 | 0.0012 | 0.0016 | 0.0039 | 0.037 |
| Burkholderia-Caballeronia-Paraburkholderia | 0.0016 | 0.0024 | 0.0001 | 0.0004 | 0.000 |
| Prevotellaceae_UCG-004 | 0.0002 | 0.0008 | 0.0014 | 0.0028 | 0.040 |
| unclassified_c__Negativicutes | 0.0013 | 0.0024 | 0.0002 | 0.0006 | 0.011 |
| Sphingomonas | 0.0010 | 0.0016 | 0.0001 | 0.0004 | 0.003 |
| Pseudomonas | 0.0005 | 0.0010 | 0.0001 | 0.0004 | 0.013 |
| Paracoccus | 0.0005 | 0.0012 | 0.0001 | 0.0004 | 0.029 |
| Brevundimonas | 0.0005 | 0.0009 | 0.0001 | 0.0004 | 0.016 |
| Nakamurella | 0.0003 | 0.0008 | 0.0000 | 0.0000 | 0.022 |
| Sediminibacterium | 0.0002 | 0.0006 | 0.0000 | 0.0000 | 0.042 |
| unclassified_f__Micrococcaceae | 0.0002 | 0.0005 | 0.0000 | 0.0000 | 0.042 |

Note: PN: S. Pullorum-negative group, PP: S. Pullorum-positive group, SD means standard deviation..
